# Subjective cognitive decline is a better marker for future cognitive decline in females than in males

**DOI:** 10.1186/s13195-022-01138-w

**Published:** 2022-12-29

**Authors:** Michael D. Oliver, Cassandra Morrison, Farooq Kamal, Jillian Graham, Mahsa Dadar

**Affiliations:** 1 Department of Psychological Science and Neuroscience, Belmont University, Nashville, TN USA; 2 Belmont Data Collaborative, Belmont University, Nashville, TN USA; 3 McConnell Brain Imaging Centre, Montreal Neurological Institute, McGill University, Montreal, QC Canada; 4 Department of Neurology and Neurosurgery, McGill University, Montreal, QC Canada; 5 Department of Psychiatry, McGill University, Montreal, QC Canada; 6 Douglas Mental Health University Institute, Montreal, QC Canada

**Keywords:** Subjective cognitive decline, Sex disparities, Cognitive change, Alzheimer’s disease

## Abstract

**Background:**

The identification of biomarkers for early detection of Alzheimer’s disease (AD) is critical to the development of therapies and interventions targeted at symptom management and tracking the pathophysiology of disease. The endorsement of subjective cognitive decline (SCD) has emerged as a potential indicator of early change in cognitive status that may be predictive of future impairment at a time when measurable declines in neuropsychological performance cannot be detected. While there are numerous findings revealing sex differences in the prevalence of AD, there is a paucity of research examining sex differences in SCD. Therefore, the goal of this project was to determine if the relationship between the endorsement of SCD and future cognitive changes differ as a function of biological sex.

**Methods:**

A sample of 3019 male and female healthy older adults (2188 without SCD, 831 with SCD), with a mean follow-up time of 5.7 years, were included from the Rush Alzheimer’s Disease Center Research Sharing Hub. Linear regressions were performed to determine group differences in baseline cognitive scores, while linear mixed-effects models were completed to determine group differences in the rate of cognitive change over time.

**Results:**

Individuals endorsing SCD had significantly lower baseline cognitive scores and increased rates of decline in all cognitive domains compared to those without SCD. Males exhibited significantly lower scores in baseline performance in global cognition, episodic memory, and perceptual speed regardless of SCD classification. Females with SCD were found to decline at significantly faster rates than both males with SCD and males and females without SCD in all cognitive domains over a maximum 15-year follow-up period.

**Conclusions:**

SCD is related to lower baseline cognitive performance and faster cognitive decline compared to those who do not endorse SCD. Females with SCD have the fastest rate of decline suggesting that SCD may be more predictive of future decline in females than in males. Targeted assessments of SCD may allow for the identification of individuals for inclusion in intervention trials, and other research studies, aiming to attenuate casual disease processes, which may ultimately aid in the mitigation of sex disparities in AD.

**Supplementary Information:**

The online version contains supplementary material available at 10.1186/s13195-022-01138-w.

## Introduction

Alzheimer’s disease (AD) is a neurodegenerative disorder characterized by pathological aggregation of the proteins amyloid-β (Aβ) and tau in the brain [52]. As Aβ plaques and neurofibrillary tau tangles form, communication between neurons is disrupted leading to atrophy, and ultimately functional impairment affecting multiple cognition domains (e.g., memory, visuospatial ability, language, and attention). Although debilitative functional changes occur with the progression of disease, it has been suggested that the pathophysiology of AD begins nearly 20 years prior to the clinical presentation of symptoms [54, 61]. Therefore, it has become critical to target AD-related biomarkers early as they may be reflective of future decline. Advancements in clinical trial research have resulted in the US Food and Drug Administration’s (FDA) recent approval of Aducanumab (Aduhelm) for AD treatment. However, due to its lack of effectiveness at improving cognitive functioning, conflicting trial results, and potential harm caused by the drug [27, 37, 55], agencies such as the European Medicines Agency (EMA) have refused to market this medication as a treatment for AD. As such, to date, there is still no uniform drug or treatment available to slow the progression of disease or reverse the disease process. For this reason, it has become increasingly important for research targeting mechanisms of early detection to help with disease prevention and to combat the deleterious effects of AD.

Research has suggested that subjective cognitive decline (SCD), or the self-reported experience of subtle changes in cognitive functioning without any measurable changes in neuropsychological test performance [25], may be a preclinical marker of AD [3]. For example, individuals who endorse SCD have an increased risk of developing AD compared to the general population [3, 49, 53]. Typically reported as increased confusion or memory loss, the prevalence of SCD among adults aged 60 and older is around 25% [50]. Therefore, SCD may prove to be an effective target for early intervention. Existing intervention studies and clinical trials targeting AD and other related dementia risk factors have revealed limited success at maintaining or improving cognitive function [1, 17, 60]. However, these interventions are often introduced after AD-related cognitive decline has already begun. This critical time window may explain why such interventions have difficulties in demonstrating effective ways of preventing and reducing cognitive decline. Although interventions have been largely unsuccessful in terms of elimination of symptoms, it has been suggested that they may be effective at ameliorating symptoms, thus improving cognitive outcomes [41]. SCD has been suggested as one of the earliest clinical indicators of AD prior to measurable cognitive decline, and its cognitive correlates align with the earliest pathological changes in AD. Therefore, explorations into SCD may allow for the improvement of early detection techniques at a critical time window prior to more pronounced atrophy and objective clinical symptoms as a consequence of disease progression.

Sex differences have also been observed in SCD, albeit findings yield inconsistent results. For example, it has been revealed that SCD in females is more strongly associated with future dementia diagnoses than in males [19]. Another study has observed that SCD in males is associated with worse performance on a measure of global cognition (Alzheimer’s Disease Assessment Scale-13) compared to females [56]. While the former suggests that SCD is associated with clinical progression in females more strongly than males, the latter indicates that SCD is associated with increased cognitive decline in males compared to females. Taken together, findings from these studies reveal that biological sex may play a role in SCD. However, further research is needed to better understand the interaction between sex and SCD. Despite evidence indicating biological sex is independently associated with AD prevalence [12, 45], cortical atrophy [15, 29], and clinical progression [14, 62], the relationship between sex, cognition, and SCD classification remains relatively unexplored. It is critical to investigate whether cognitive decline observed in people with SCD differs as a function of biological sex. Such an exploration may result in a better understanding of whether females endorsing SCD have different cognitive trajectories subjecting them to greater decline and higher prevalence of AD compared to males. To examine this relationship, we investigated sex differences in cognition in a sample of female and male healthy older adults with and without SCD. The importance of examining sex differences in both groups is to ensure that the change over time is specific to those with SCD and not simply what occurs in healthy “normal” aging in this sample. This design allows us to determine whether SCD is predictive of future cognitive decline and if this association differs by biological sex.

## Methods

### Participants

Data used in preparation of this article were obtained from the RADC Research Resource Sharing Hub (www.radc.rush.edu). Participants provided informed written consent to participate in one of three cohort studies on aging and dementia: (1) Minority Aging Research Study [4], (2) Rush Alzheimer’s Disease Center Clinical Core [51], or (3) the Rush Memory and Aging Project [10]. Demographic and clinical characteristics by cohort are provided in Table 1. More information about each cohort study design can be accessed online through the RADC Research Resource Sharing Hub (https://www.radc.rush.edu/docs/parentStudyDesigns.htm).

**Table 1:** Demographic information for each study included in this paper

	CORE (*n* = 254)	LATC (*n* = 148)	MAP (*n* = 1211)	MARS (*n* = 519)	ROS (*n* = 887)
Baseline age	72.1 ± 6.0	70.1 ± 6.1	79.0 ± 7.1	72.5 ± 5.7	74.3 ± 6.8
Education	15.0 ± 3.0	9.8 ± 4.9	15.2 ± 3.3	15.1 ± 3.5	18.6 ± 3.4
Baseline SCD +	68 (27%)	49 (33%)	354 (29%)	141 (27%)	219 (25%)
Females	210 (83%)	115 (78%)	929 (77%)	407 (78%)	634 (72%)
Mean follow-up	4.8 ± 2.9	2.4 ± 1.2	5.4 ± 3.7	5.3 ± 3.6	6.5 ± 4.1
Baseline global cognition	0.07 ± 0.45	− 0.20 ± 0.49	0.31 ± 0.43	0.09 ± 0.43	0.40 ± 0.41
Baseline episodic memory	0.27 ± 0.50	− 0.04 ± 0.53	0.33 ± 0.49	0.19 ± 0.45	0.43 ± 0.46
Baseline semantic memory	− 0.04 ± 0.79	− 0.08 ± 0.74	0.35 ± 0.62	0.11 ± 0.65	0.36 ± 0.62
Baseline perceptual speed	0.11 ± 0.65	− 0.13 ± 0.77	0.24 ± 0.73	0.06 ± 0.71	0.43 ± 0.76
Baseline visuospatial abilities	− 0.16 ± 0.74	− 0.16 ± 0.74	0.37 ± 0.63	− 0.12 ± 0.72	0.41 ± 0.59
Baseline working memory	− 0.13 ± 0.74	− 0.77 ± 0.66	0.27 ± 0.70	0.02 ± 0.70	0.31 ± 0.73

Scores are presented as mean ± standard deviation or number of sample and percentage of population. *SCD* + cognitively healthy older adults with subjective cognitive decline. *CORE* Clinical CORE Study, *LATC* Latino CORE Study, *MAP* Memory and Aging Project, *MARS* Minatory Aging Research Study, *ROS* Religious orders study

Participant inclusion criteria for this specific study were as follows: (1) cognitively normal/healthy (NC/SCD −) status at their baseline visit (e.g., no mild cognitive impairment, MCI), (2) no report of stroke, (3) had completed at least two cognitive assessments, (4) completed the questionnaire assessing memory complaints, and (5) at least 55 years of age at baseline. A clinical diagnosis of cognitive status was completed using a three-stage process including computer scoring of cognitive tests, clinical judgment by a neuropsychologist, and diagnostic classification by a clinician based on criteria of the joint working group of the National Institute on Aging and the Alzheimer’s Association (NIA-AA) [39]. Our two samples included a total of 3019 healthy older adult participants with a mean follow-up time of 5.7 years (with a total of 24,689 follow-ups available for analysis, hereafter referred to as Time From Baseline). The healthy control (NC/SCD −) sample (*N* = 2188) contained 528 Males and 1660 Females. The SCD sample (*N* = 831) contained 196 Males and 635 Females. Both samples had an equal sex distribution (24% male, 76% female).

Consistent with previous work investigating memory concerns in the RUSH cohort, subjective cognitive decline was defined based on two questions examining memory complaints [2, 5, 20]. Participants were asked, “About how often do you have trouble remembering things?” and “Compared to 10 years ago, would you say that your memory is much worse, a little worse, the same, a little better, or much better?” Both questions were scored using a scale of 1 to 5 with 5 being often/worse and 1 being never/much better. Following past research and the RUSH recommendations, if the participants scored 8–10 on these two questions, they were classified as having memory complaints [2], reported as subjective cognitive decline (SCD +) in this study.

### Cognitive assessment

All participants were administered a battery of neuropsychological tests including 19 tests selected to assess five cognitive domains and a measure of overall global cognitive function [5, 58]. There were seven tests of episodic memory (immediate and delayed recall of Story A of the Wechsler Memory Scale-Revised; immediate and delayed recall of the East Boston Story; Word List Memory, Recall and Recognition), three tests of semantic memory (Verbal Fluency; Boston Naming; Reading Test), three tests of working memory (Digit Span forward and backward; Digit Ordering), four tests of perceptual speed (Symbol Digit Modalities Test; Number Comparison; two indices from a modified version of the Stroop Test), and two tests of visuospatial ability (Line Orientation; Progressive Matrices). Composite measures of each domain were used in analyses, as well as a global composite of all tests. To create each composite score, individual tests were converted to *z*-scores, using the mean and standard deviation from the combined cohort at baseline, and *z*-scores for the relevant tests were averaged. An individual’s standard performance across all 19 of these tests was averaged to create a measure of global cognitive function [30]. More information for the specific tests used for each category can be obtained from https://www.radc.rush.edu/.

### Statistical analysis

Analyses were performed using “R” software version 4.0.5. Independent sample *t*-tests were completed on age and education. Multiple comparisons were corrected for using Bonferroni correction. Differences in baseline cognitive scores between male and females with and without SCD were examined using linear regressions (“lm,” package “stats” in “R”). Differences in rates of change in cognition between males and females were investigated using linear mixed effects models (“lmer,” package “lme4” in R [7]. These models examined the association between each cognitive domain (i.e., global, episodic memory, semantic memory, perceptual speed, working memory, and visuospatial abilities), SCD classification (i.e., SCD + and SCD −), and sex (i.e., male and female). All models were corrected for multiple comparisons using false discovery rate (FDR) [9], *p*-values were reported as raw values with significance, then determined by FDR correction. All continuous values were *z*-scored within the population prior to the analyses.

Bonferroni correction was completed on demographic information because there is no expectation of correlation between those variables (e.g., age and sex). Therefore, using Bonferroni correction with the assumption of independence between tests is valid. However, due to presence of interdependency between the regression and mixed effects models performed, FDR was completed. In other words, the outputs from the regressions are going to be, by nature, associated and therefore Bonferroni would over correct (increase type 2 error) in these cases whereas FDR would not.

To investigate the influence of sex on baseline cognitive scores by SCD classification, linear regressions for each cognitive domain were completed. The interaction of interest was SCD:MaleSex, to examine if baseline cognitive scores differed between males and females in each group. The models also included years of education and age at baseline (Age_bl) as covariates.

1 $$\begin{array}{c}Cognitive\;Score \sim SCD:Sex + SCD + Sex + Age\_bl + Education \\ R\;syntax: lm(Cognitive\;Score \sim Sex*SCD + Age\_bl + Education)\end{array}$$

For the longitudinal analysis, the categorical variables of interest were sex (i.e., male vs. female), contrasting the males against the females and SCD classification (i.e., SCD + vs SCD −), contrasting SCD + against SCD − . The models also included Time From Baseline, years of education, and age at baseline (*Age_bl*) as covariates. The interactions of interest were Sex:TimeFromBaseline, SCD:TimeFromBaseline, and Sex:SCD:TimeFromBaseline to examine if change over time differed between males and females within each group. To investigate whether there were significant differences in SCD + and SCD − males, the models were repeated a second time, using NC males as the reference. Participant ID was included as a categorical random effect to account for repeated measures of the same participant.

2 $$\begin{array}{c}Cognitive\;Score \sim Sex:TimeFromBaseline:SCD + Sex:TimeFromBaseline +\\ TimeFromBaseline:SCD + Sex:SCD + Sex + TimeFromBaseline + SCD + Age\_bl + Education + (1|ID)\\ R\;syntax: lmer(Cognitive Score \sim Sex*TimeFromBaseline*SCD + Age\_bl + Education + (1|ID))\end{array}$$

Residuals and random effects coefficients were inspected to ensure that the assumptions of the linear mixed effects models were met. R syntax for the models has been displayed to enable replication of the models.

## Results

### Demographics and baseline cognitive scores

In both SCD groups, males had higher education than females (SCD + : *t* = 4.30, *p* < 0.001; SCD − :* t* = 4.23, *p* < 0.001). Age did not significantly differ between SCD + males and females (*t* = 1.96) or SCD − males and females (*t* = 1.55). Table 1 presents the demographic information for each cohort included in this study.

**Fig. 1:**
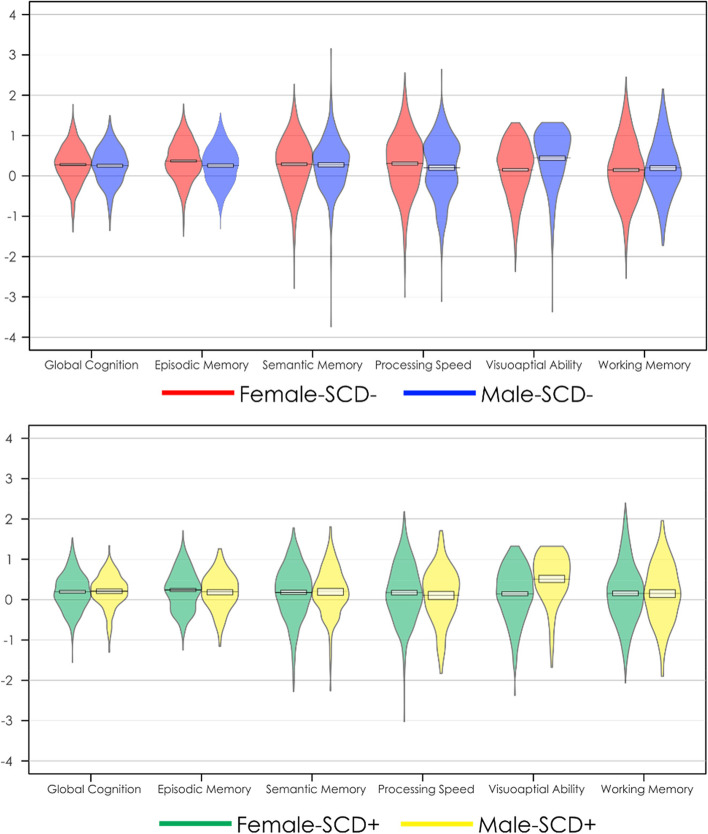
Baseline cognitive differences in each domain for females and males with and without subjective cognitive decline. **Top image represents** baseline cognitive scores with mean and standard deviation for healthy older adults without subjective cognitive decline (SCD −). **Bottom image represents** baseline cognitive scores with mean and standard deviation for healthy older adults with subjective cognitive decline (SCD +)

Figure 1a plots baseline cognitive scores for female and male SCD − participants. Figure 1b plots baseline cognitive scores for female and male SCD + participants. Table 2 provides the outputs for the baseline linear regression models. Increased age was associated with lower cognitive scores at baseline in all cognitive domains (*t* belongs to [− 8.80 to − 15.19], *p* < 0.001) except visuospatial ability and working memory. On the other hand, increased education was associated with higher cognitive scores in all domains (*t* belongs to [18.33–30.15], *p* < 0.001). Males had lower global cognition, episodic memory, and perceptual speed (*t* belongs to [− 4.13 to − 7.50], *p* < 0.01), but higher visuospatial scores (*t* = 7.50, *p* < 0.001) compared to females at baseline, regardless of SCD classification. SCD classification was associated with lower scores in baseline global cognition, episodic memory, semantic memory, perceptual speed (*t* belongs to [− 4.58 to − 2.30, *p* < 0.05), but not visuospatial abilities or working memory. Further, none of the SCD by sex interactions were significant.

**Table 2:** Linear regression outputs for baseline data

	Global cognition	Episodic memory	Semantic memory	Perceptual speed	Visuospatial abilities	Working memory
Age at baseline	***β* = − 0.13**	***β* = − 0.12**	***β* = − 0.13**	***β* = − 0.24**	*β* = − 0.02	*β* = 0.02
	***SE* = 0.01**	***SE* = 0.01**	***SE* = 0.01**	***SE* = 0.02**	*SE* = 0.02	*SE* = 0.01
	***t* = − 10.82**	***t* = − 10.47**	***t* = − 8.80**	***t* = − 15.19**	*t* = − 0.99	*t* = 1.67
	***p* < .001**	***p* < .001**	***p* < .001**	***p* < .001**	*p* = .32	*p* = 0.94
Male sex	***β* = − 0.13**	***β* = − 0.22**	*β* = − 0.08	***β* = − 0.22**	***β* = 0.32**	*β* < − 0.01
	***SE* = 0.03**	***SE* = 0.03**	*SE* = 0.04	***SE* = 0.04**	***SE* = 0.04**	*SE* = 0.04
	***t* = − 4.13**	***t* = − 7.24**	*t* = − 2.06	***t* = − 5.52**	***t* = 7.50**	*t* = − 0.25
	***p* < .001**	***p* < .001**	*p* = .04	***p* < .001**	***p* < .001**	*p* = .80
Education	***β* = 0.33**	***β* = 0.22**	***β* = 0.25**	***β* = 0.30**	***β* = 0.30**	***β* = 0.25**
	***SE* = 0.01**	***SE* = 0.01**	***SE* = 0.01**	***SE* = 0.01**	***SE* = 0.02**	***SE* = 0.01**
	***t* = 30.15**	***t* = 21.03**	***t* = 18.33**	***t* = 21.05**	***t* = 20.18**	***t* = 20.48**
	***p* < .001**	***p* < .001**	***p* < .001**	***p* < .001**	***p* < .001**	***p* < .001**
SCD classification	***β* = − 0.08**	***β* = − 0.13**	***β* = − 0.09**	***β* = − 0.09**	*β* = 0.03	*β* = 0.03
	***SE* = 0.03**	***SE* = 0.03**	***SE* = 0.04**	***SE* = 0.04**	*SE* = 0.04	*SE* = 0.03
	***t* = − 2.80**	***t* = − 4.58**	***t* = − 2.39**	***t* = − 2.30**	*t* = 0.66	*t* = 0.85
	***p* = .005**	***p* < *.*001**	***p* = .017**	***p* = .021**	*p* = .51	*p* = .39
SCD classification:Male sex	*β* = 0.01	*β* = 0.05	*β* = 0.01	*β* < − 0.01	*β* = 0.05	*β* = − 0.08
	*SE* = 0.04	*SE* = 0.06	*SE* = 0.06	*SE* = 0.03	*SE* = 0.08	*SE* = 0.07
	*t* = 0.24	*t* = 0.94	*t* = 0.10	*t* = − 0.06	*t* = 0.59	*t* = − 1.25
	*p* = 0.81	*p* = 0.35	*p* = .92	*p* = .95	*p* = .56	*p* = .21

Bolded values are results that remained significant after FDR correction

### Cognitive change

**Fig. 2:**
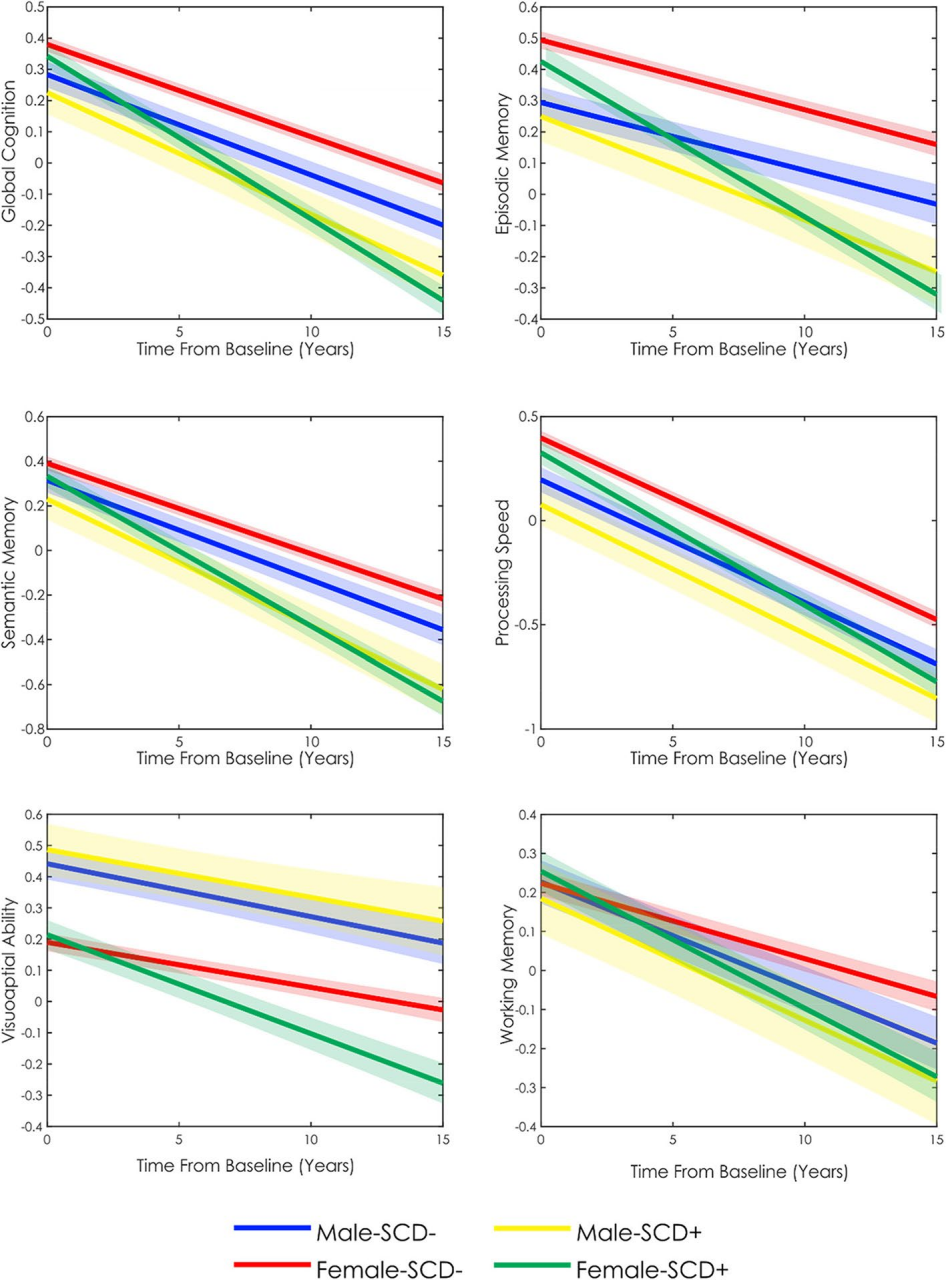
Longitudinal cognitive change over time in females and males with and without subjective cognitive decline. SCD − , older adults without subjective cognitive decline. SCD + , older adults with subjective cognitive decline

Figure 2 shows the mixed effects model predictions of cognitive scores over time for each cognitive domain by sex and SCD classification. Table 3 provides the estimates for the mixed effects model. For all cognitive domains, Time From Baseline (*t* belongs to [− 11.60 to − 53.54], *p* < 0.001) and increased age at baseline (*t* belongs to [− 4.08 to − 22.48], *p* < 0.001) were associated with lower cognitive performance. Increased education was associated with increased performance in all cognitive domains (*t* belongs to [17.26–25.40], *p* < 0.001). All results remained significant after FDR correction.

**Table 3:** Linear mixed effects output for longitudinal data

	Global cognition	Episodic memory	Semantic memory	Perceptual speed	Visuospatial abilities	Working memory
Age at baseline	***β* = − 0.26**	***β* = − 0.25**	***β* = − 0.23**	***β* = − 0.32**	***β* = − 0.09**	***β* = − 0.06**
	***SE* = 0.01**	***SE* = 0.01**	***SE* = 0.01**	***SE* = 0.01**	***SE* = 0.01**	***SE* = 0.01**
	***t* = − 19.58**	***t* = − 19.31**	***t* = − 16.96**	***t* = − 22.48**	***t* = − 6.78**	***t* = − 4.08**
	***p* < .001**	***p* < .001**	***p* < .001**	***p* < .001**	***p* < .001**	***p* < .001**
Education	***β* = 0.34**	***β* = 0.24**	***β* = 0.24**	***β* = 0.29**	***β* = 0.29**	***β* = 0.31**
	***SE* = 0.01**	***SE* = 0.01**	***SE* = 0.01**	***SE* = 0.01**	***SE* = 0.01**	***SE* = 0.01**
	***t* = 25.40**	***t* = 17.98**	***t* = 17.26**	***t* = 20.43**	***t* = 21.32**	***t* = 21.52**
	***p* < .001**	***p* < .001**	***p* < .001**	***p* < .001**	***p* < .001**	***p* < .001**
SCD classification	***β* = − 0.24**	***β* = − 0.27**	***β* = − 0.23**	***β* = − 0.17**	***β* = − 0.09**	*β* = − 0.06
	***SE* = 0.04**	***SE* = 0.03**	***SE* = 0.04**	***SE* = 0.04**	***SE* = 0.03**	*SE* = 0.04
	***t* = − 6.71**	***t* = − 7.88**	***t* = − 6.43**	***t* = − 4.48**	***t* = − 2.28**	*t* = − 1.61
	***p* < .001**	***p* < .001**	***p* < .001**	***p* < .001**	***p* = .022**	*p* = .11
Male sex	***β* = − 0.18**	***β* = − 0.26**	***β* = − 0.12**	***β* = − 0.24**	***β* = 0.32**	*β* = *− *0.05
	***SE* = 0.04**	***SE* = 0.04**	***SE* = 0.04**	***SE* = 0.04**	***SE* = 0.04**	*SE* = 0.04
	***t* = − 4.69**	***t* = − 7.09**	***t* = − 3.05**	***t* = − 5.88**	***t* = 8.33**	*t* = *− *1.18
	***p* < .001**	***p* < .001**	***p* = .002**	***p* < .001**	***p* < .001**	*p* = .24
TimeFromBaseline	***β* = − 0.19**	***β* = − 0.12**	***β* = − 0.20**	***β* = − 0.28**	***β* = − 0.08**	***β* = − 0.10**
	***SE* < 0.01**	***SE* < 0.01**	***SE* < 0.01**	***SE* < 0.01**	***SE* < 0.01**	***SE* < 0.01**
	***t* = − 35.14**	***t* = − 19.02**	***t* = − 34.50**	***t* = − 53.54**	***t* = − 11.60**	***t* = − 17.43**
	***p* < .001**	***p* < .001**	***p* < .001**	***p* < .001**	***p* < .001**	***p* < .001**
Male sex: SCD classification	*β* = 0.09	*β* = 0.14	*β* = 0.06	*β* = 0.01	*β* = 0.15	*β* = − 0.02
	*SE* = 0.07	*SE* = 0.07	*SE* = 0.07	*SE* = 0.07	*SE* = 0.08	*SE* = 0.08
	*t* = 1.27	*t* = 1.96	*t* = 0.75	*t* = 0.17	*t* = 2.10	*t* = − 0.22
	*p* = .20	*p* = .05	*p* = .45	*p* = .86	*p* = .036	*p* = .83
SCD: TimeFromBaseline (females)	***β* = − 0.14**	***β* = − 0.15**	***β* = − 0.13**	***β* = − 0.07**	***β* = − 0.09**	***β* = − 0.08**
	***SE* = 0.01**	***SE* = 0.01**	***SE* = 0.01**	***SE* = 0.01**	***SE* = 0.01**	***SE* = 0.01**
	***t* = − 12.85**	***t* = − 11.88**	***t* = − 11.49**	***t* = − 7.07**	***t* = − 7.02**	***t* = − 7.20**
	***p* < .001**	***p* < .001**	***p* < .001**	***p* < .001**	***p* < .001**	***p* < .001**
Male sex: TimeFromBaseline	*β* = *− *0.02	*β* < 0.01	*β* = *− *0.02	*β* < *− *0.01	*β* = *− *0.01	***β* = *− *0.04**
	*SE* < 0.01	*SE* < 0.01	*SE* = 0.01	*SE* < 0.01	*SE* = 0.01	***SE* = 0.01**
	*t* = *− *1.73	*t* = 0.25	*t* = *− *1.78	*t* = *− *0.37	*t* = *− *1.02	***t* = *− *3.72**
	*p* = .08	*p* = .80	*p* = .07	*p* = .71	*p* = .30	***p* < .001**
MaleSex: SCD classification: TimeFromBaseline	***β* = 0.10**	***β* = 0.09**	***β* = 0.07**	***β* = 0.06**	***β* = 0.10**	***β* = 0.06**
	***SE* = 0.02**	***SE* = 0.02**	***SE* = 0.02**	***SE* = 0.02**	***SE* = 0.03**	***SE* = 0.02**
	***t* = 4.65**	***t* = 3.56**	***t* = 3.22**	***t* = 2.96**	***t* = 4.00**	***t* = 2.84**
	***p* < .001**	***p* < .001**	***p* = .001**	***p* = .003**	***p* < .001**	***p* = .004**
**Contrasts**						
SCD classification: TimeFromBaseline (males)	***β* = *− *0.04**	***β* = *− *0.06**	***β* = *− *0.06**	*β* = *− *0.01	*β* < 0.01	*β* = *− *0.02
	***SE* = 0.02**	***SE* = 0.02**	***SE* = 0.02**	*SE* = 0.02	*SE* = 0.02	*SE* = 0.02
	***t* = *− *2.25**	***t* = *− *2.97**	***t* = *− *3.12**	*t* = *− *0.87	*t* = 0.4	*t* = *− *1.00
	***p* = .024**	***p* = .003**	***p* = .001**	*p* = .38	*p* = .68	*p* = .32

Bolded values are results that remained significant after FDR correction. The additional contrast was completed to examine the difference in rate of change for male SCD + vs. male SCD −

The main effect of SCD was significant for all domains except working memory (*t* belongs to [− 2.28 to − 7.88], *p* < 0.05). The main effect of male sex was significant for all domains except working memory. Males exhibited lower overall performance in global cognition, episodic memory, semantic memory, and perceptual speed (*t* belongs to [− 3.05 to − 7.09], *p* < 0.005), but higher overall performance in visuospatial abilities (*t* = 8.33, *p* < 0.001). Male by SCD Classification was not significant for any cognitive domain, and the male by Time Fom Baseline year was only significant for working memory (*t* =  − 3.72, *p* < 0.001). The SCD classification by Time From Baseline interaction was significant for all cognitive domains for females (*t* belongs to [− 7.02 to − 12.85], *p* < 0.001), indicating that females with SCD had increased rates of decline in all domains compared to SCD − . In comparison, for males, the SCD classification by Time From Baseline interaction was significant for global cognition, episodic, and semantic memory (*t* belongs to [− 2.25 to − 3.12], *p* < 0.05). The three-way interaction between male sex, SCD classification, and Time From Baseline was significant for all cognitive domains (*t* belongs to [2.84–4.65], *p* < 0.005). Taken together, these results suggest that SCD + females decline at significantly faster rates than SCD − females in all cognitive domains (*t* belongs to [− 7.02 to − 12.85], *p* < 0.001), whereas SCD + males decline at faster rates than SCD − males only in global cognition, episodic memory, and semantic memory (*t* belongs to [− 2.25 to 3.12], *p* < 0.05). While SCD − males and females do not differ in terms of rate of cognitive decline in any cognitive domain except for working memory, in which males exhibit a faster decline, SCD + females decline at significantly faster rates than SCD + males in all cognitive domains (Fig. 2, Table 2).

It should be noted that all models were repeated including study cohort as a random categorical effect (1|study) and with additional interactions for Age:TimeFromBaseline as well as Education:TimeFromBaseline. These additional analyses were completed to ensure results were not driven by differences in either cohort demographics or in how age and education influence change over time between the sexes. These models produced similar results in terms of effect size and significance reflecting an insignificant role of cohort, age by Time From Baseline, and education and Time From Baselineon the current findings.

For a more conservative assessment, we also repeated the models including Time From Baseline as a random slope: (1 + TimeFromBaseline| ID). To achieve slopes that accurately represent the data, a large number of follow-ups for each person is required. More specifically, based on the work by Wright et al. [59] to obtain estimated slopes that have a high correlation (> 0.8) with the true underlying slopes, 8 timepoints per person are needed. Therefore, participants were included in this analysis if they had at least 8 follow-up visits. Our sample was reduced to 1305 participants with 16,335 timepoints. Results for this analysis are presented in Supplementary Table 1. Almost all results remained the same in terms of effect size and significance. For episodic memory, the three-way interaction between male sex, SCD classification, and Time From Baseline was no longer significant. Secondly, the SCD classification by Time From Baseline interaction for males was also no longer significant for global cognition, episodic, and semantic memory. That is, males with and without SCD did not differ in their rate of change over time in any cognitive domain when controlling for Time From Baseline using a random slope. To ensure that these differences are not caused by the reduction in our sample size due to the additional criterion of 8 follow-up visits per participant, the models were repeated in this subset without the term (1+TimeFromBaseline|ID). The results were similar to those of the full sample in terms of effect size and significance.

## Discussion

Previous findings have suggested that cognitive functioning, including rate of cognitive decline, may differ between males and females (e.g., [32]. However, there is limited research examining sex differences in people who may be at the earliest stages of cognitive decline, those with SCD. This limited understanding of how sex may influence cognitive decline in SCD limits the ability to have targeted interventions and therapies to help prevent cognitive decline due to MCI or dementia. Therefore, the current study aimed to elucidate sex disparities in the trajectory of cognitive decline to aid in a better understanding of techniques for early detection and disease mitigation. In our sample of 3019 cognitively unimpaired older adults, the rate of change in cognitive performance varied between males and females in people with SCD. Specifically, males exhibited significantly lower baseline performance in global cognition, episodic memory, and perceptual speed but higher performance in visuospatial abilities. The three-way interaction between Sex, SCD classification, and Time From Baseline was also significant, revealing that SCD + females decline at a significantly faster rate than SCD + males in all cognitive domains. When examining the interaction effect of SCD classification and Time From Baseline in the longitudinal model, SCD + males exhibited significantly lower overall performance in global cognition, episodic, and semantic memory compared to SCD − males, while SCD + females exhibited significantly lower performance in all cognitive domains compared to SCD − females. Our results reveal that (1) people with SCD have both lower baseline cognition and an increased rate of decline compared to people without SCD, and (2) SCD in females may be more predictive of future cognitive decline than in males, which may help explain sex disparities in cognitive decline.

There is mounting evidence suggesting that sex differences exist in both normal cognition and dementia. For example, a recent cohort study of over 26,000 participants reported cognitively unimpaired females to have greater global cognition, executive function, and memory compared to males [32]. Similarly, in our study, a main effect of sex was observed for all cognitive domains except working memory, demonstrating that regardless of SCD status, females tend to score higher on neuropsychological assessments in several domains compared to males, whereas males score higher in visuospatial ability then females. Levine and colleagues [32] also observed that cognitively unimpaired females had an increased rate of decline compared to males. Additionally, several other studies have suggested that although females may score higher at baseline, they may be subject to faster cognitive decline compared to males [21, 23, 34]. This increased rate of decline in females may contribute to the sex disparities that exist in prevalence of AD [8, 35]. In the present study, females were not observed to have an increased rate of decline in the SCD − group compared to males. Rather, sex differences in cognitive decline were observed only in SCD + group. SCD + females experienced steeper declines in cognitive performance compared to SCD + males, and SCD − males and females, in all domains. That is, although SCD − females are shown to consistently have the highest cognitive performance over time compared to SCD − males (except in visuospatial abilities), and SCD + males and females, the introduction of SCD ( +), negatively affects this relationship, which may be indicative of a more rapid trajectory of cognitive decline.

While previous research has examined sex differences in SCD status, the results are limited to smaller samples and cross-sectional data [56] as well as only examining subsequent dementia (and AD) conversion [19, 47], global cognition, and instrumental activities of daily living [47] and not rate of cognitive change. Previously, females with SCD have been reported to exhibit lower overall baseline global cognition, enhanced memory, and similar performance on executive functioning and semantic memory tasks compared to males with SCD [56]. The current study expanded on this work by examining a large sample with longitudinal assessments of cognitive performance in order to further explore sex discrepancies. The longitudinal assessment is particularly important given that at baseline, females exhibited enhanced performance than males in most domains (except semantic memory and visuospatial ability), whereas females with SCD + had an increased rate of decline in all domains (i.e., global cognition, episodic memory, semantic memory, perceptual speed, visuospatial ability, and working memory). Furthermore, in the current study, we focused on examining sex differences in the trajectory of cognitive decline over time, rather than the relationship between worry and conversion in individuals with SCD. Future research should examine whether sex differences are present in the relationship between SCD and worry with cognitive decline.

Given that SCD (SCD +) has been linked to increased risk for MCI and AD [24], those who endorse these subjective cognitive complaints are at greater risk for subsequent cognitive decline compared to those that do not (e.g., [44]). In the current study, both male and female SCD + participants had increased rate of decline compared to SCD − participants, supporting the notion that SCD is indicative of future decline [24, 28]. However, SCD + males only had increased rates of decline in global cognition, episodic memory, and semantic memory compared to SCD − males, whereas SCD + females decline at significantly faster rates than SCD − females in all cognitive domains. When employing the more conservative approach, SCD + males did not have increased rates of decline in any domain compared to SCD − males. With the presence of sex differences in SCD + (i.e., SCD + females exhibiting increased rates of change in all cognitive domains compared to SCD + males), our findings suggest that the relationship between SCD and future cognitive changes may be more predictive of cognitive decline in females compared to males. That is, females reporting SCD may be more likely to experience substantial cognitive changes compared to males with SCD. These findings suggest that waning cognitive abilities may have the potential to be captured early, particularly in females, with SCD + individuals detecting subtle cognitive changes prior to objective testing. Previous studies have observed that females tend to self-report cognitive changes more than males [38]. Combining our findings with the increased reports in females relative to males may be indicative of either greater changes or better perception of cognitive changes in females. As such, the relationship between SCD and cognition may be stronger in females.

Findings from this study have important implications for interventions and therapies designed to target cognitive decline and dementia prevention. For example, risk factors such as midlife hypertension, midlife obesity, diabetes, physical inactivity, smoking, depression, and low education are all modifiable factors contributing to 1/3^rd^ of all AD cases [46]. However, several of these AD risk factors disproportionately affect females. For example, both lower educational attainment, as well as psychiatric disorders such as depression, are more prevalent in females [22]. Additionally, blood pressure is observed to be higher in males early in life, whereas females have a steeper increase in blood pressure that continues throughout the life compared to males [26]. This prevalence of higher mid-life blood pressure in females is associated with a greater risk for the development of dementia compared to males [11]. Other factors specific to females such as preeclampsia, menopause, and hypertensive pregnancy disorders also have negative impacts on the cardiovascular system and cognition [16, 40]. These risk factors, paired with explanations such as higher life expectancy [18], lower cognitive reserve, and faster rates of functional and structural deterioration [31] in females compared to males have all led previous literature to reveal female sex to be a significant risk factor for AD [8, 35]. The current study supplements the existing literature by revealing that in the earliest potential stage of the AD-trajectory prior to measurable cognitive decline (i.e., preclinical-AD or SCD), females also exhibit steeper declines in all cognitive domains over time compared to males. As such, directing therapies and interventions toward risk factors that have increased incidence in females may help reduce the prevalence of dementia in these individuals. Our findings suggest that SCD may be a critical indicator of subsequent cognitive decline in females, and therapeutic interventions may wish to target this population to better elucidate sex disparities in cognitive change over time.

One limitation of the current work is the use of only two questions to determine SCD status. Previous research has shown that different questionnaires used to determine SCD status results in different cognitive trajectories and atrophy patterns [42] as well as different patterns of white matter hyperintensity burden [43]. Therefore, it is thus possible that the use of different questionnaires may target specific declines in males vs. females and improve the relationship between SCD and cognition in males. Future research should explore this relationship. Females also have increased risk factors that influence vascular components. The resulting pathological changes due to vascular damage, such as white matter hyperintensities which are known to be associated with cognitive decline and conversion to dementia [13], may be higher in females with SCD. Future research should examine the association between sex and SCD status on atrophy and white matter hyperintensities. Another limitation is that there are several factors that may influence the endorsement of SCD (e.g., depression, worry, and personality). Several studies have demonstrated that females are much more likely than males to experience depression [48] and develop anxiety disorders due to excessive worrying [36], and they tend to have distinctly different personalities compared to males [57]. The fact that sex differences are prevalent in these domains affecting SCD may potentially contribute to disparities in the experience and subsequent endorsement of SCD. Although findings are inconsistent on the potential effects of these factors, they may play a role in either contributing to, or exacerbating, the association of SCD with the risk of decline and/or dementia [19, 33]. Future research should further examine the influence of various covariates on SCD to determine its biological basis and whether these factors mediate the relationship between SCD and cognitive decline.

## Conclusion

The current study compared cognitively unimpaired males and females with and without SCD to demonstrate that sex differences influence rate of change in cognitive performance over time. Our findings suggest that while both males and females with SCD have lower baseline cognitive scores compared to those without SCD, SCD may be more predictive of future decline in females than in males. These findings have implications for clinical and research settings where future prediction of cognitive decline are examined. For example, our findings suggest that when an individual presents with SCD, they may be at a greater risk for cognitive decline compared to those who do not endorse SCD. This knowledge may allow for targeted assessments of SCD in clinical and research settings to identify those with SCD for inclusion in research studies and trials aiming to better understand early changes associated with cognitive decline. Furthermore, these findings should be considered when developing interventions to slow progression of cognitive decline particularly in females, with the overall goal of lowering the rate of decline and conversion to dementia.

## Supplementary Information

**Additional file 1: Supplemental Table 1.** Linear mixed effects model including time from baseline as a random slope.

## Data Availability

Data used in preparation of this study were obtained from the RADC Research Resource Sharing Hub and are available from the RADC database (www.radc.rush.edu) upon registration and compliance with the data usage agreement.
