# Supplementary material for: Subjective cognitive decline is a better marker for future cognitive decline in females than in males
Source: Alzheimers Res Ther. 2022 Dec 29;14:197. doi: 10.1186/s13195-022-01138-w (PMC9798694; doi:10.1186/s13195-022-01138-w)
Supplement: Supplementary file 1 — Additional file 1: Supplemental Table 1. Linear mixed effects model including time from baseline as a random slope. [file 13195_2022_1138_MOESM1_ESM.docx]

|  | Global Cognition | Episodic Memory | Semantic Memory | Perceptual Speed | Visuospatial Abilities | Working Memory |
| --- | --- | --- | --- | --- | --- | --- |
| Age at Baseline | ***β* = -0.17**  ***SE =* 0.02**  ***t* = -9.37 *p*<.001** | ***β* = -0.14**  ***SE =* 0.02**  ***t* = -8.77 *p*<.001** | ***β* = -0.16**  ***SE =* 0.02**  ***t* = -8.86 *p*<.001** | ***β* = -0.24**  ***SE =* 0.02**  ***t* = -11.03 *p*<.001** | ***β =* -0.09**  ***SE =* 0.02**  ***t* = -3.97 *p*<.001** | ***β =* 0.06**  ***SE =* 0.02**  ***t* = -2.87 *p*<.001** |
| Education | ***β* = 0.28**  ***SE =* 0.02**  ***t* = 15.55 *p*<.001** | ***β* = 0.19**  ***SE =* 0.02**  ***t* = 11.44 *p*<.001** | ***β* = 0.17**  ***SE =* 0.02**  ***t* = 9.33 *p*<.001** | ***β* = 0.25**  ***SE =* 0.02**  ***t* = 11.29 *p*<.001** | ***β* = 0.25**  ***SE =* 0.02**  ***t* = 12.01 *p*<.001** | ***β* = 0.26**  ***SE=* 0.02**  ***t* = 11.80 *p*<.001** |
| SCD Classification | ***β* = -0.27**  ***SE =* 0.05**  ***t* = -5.33 *p<*.001** | ***β* = -0.28**  ***SE =* 0.05**  ***t* = -5.34 *p<*.001** | ***β* = -0.29**  ***SE =* 0.05**  ***t* = -5.82 *p<*.001** | ***β* = -0.25**  ***SE =* 0.06**  ***t* = -4.32 *p<.*001** | ***β* = -0.12**  ***SE =* 0.05**  ***t* = -2.38 *p*=.017** | *β* = -0.06  *SE =* 0.05  *t* = -1.00 *p*=.32 |
| Male Sex | ***β* = -0.18**  ***SE =* 0.05**  ***t* = -3.36 *p=*.001** | ***β* = -0.23**  ***SE =* 0.05**  ***t* = -4.31 *p*<.001** | ***β* = -0.15**  ***SE =* 0.05**  ***t* = -2.87 *p<*.001** | ***β* = -0.30**  ***SE =* 0.06**  ***t* = -5.02 *p*<.001** | ***β* = 0.33**  ***SE =* 0.05**  ***t* = 6.18 *p*<.001** | *β =* -0.05  *SE =* 0.06  *t* = -0.80 *p=*.43 |
| TimeFromBaseline | ***β* = -0.21**  ***SE =* 0.01**  ***t* = -13.84 *p*<.001** | ***β* = -0.14**  ***SE =* 0.02**  ***t* = -7.67 *p*<.001** | ***β* = -0.22**  ***SE =* 0.01**  ***t* = -14.79 *p*<.001** | ***β* = -0.31**  ***SE =* 0.01**  ***t* = -23.99 *p*<.001** | ***β* = -0.09**  ***SE =* 0.01**  ***t* = -7.44 *p*<.001** | ***β* = -0.10**  ***SE =* 0.01**  ***t* = -9.65 *p*<.001** |
| Male Sex: SCD Classification | *β* = 0.16  *SE =* 0.09  *t* = 1.66 *p*=.096 | *β* = 0.21  *SE =* 0.10  *t* = 2.01 *p*=.044 | *β* = -0.20  *SE =* 0.10  *t* = 1.98 *p*=.047 | *β* = 0.05  *SE =* 0.11  *t* = 0.45 *p*=.65 | *β* = 0.15  *SE =* 0.10  *t* = 1.48 *p*=.13 | *β* = -0.01  *SE =* 0.10  *t* = -0.09 *p*=.93 |
| SCD: TimeFromBaseline (females) | ***β* = -0.16**  ***SE =* 0.03**  ***t* = -5.61 *p*<.001** | ***β* = -0.18**  ***SE =* 0.03**  ***t* = -5.25 *p*<.001** | ***β* = -0.17**  ***SE =* 0.03**  ***t* = -5.87 *p*<.001** | ***β* = -0.09**  ***SE =* 0.02**  ***t* = -3.60 *p*<.001** | ***β* = -0.11**  ***SE =* 0.02**  ***t* = -4.78 *p*<.001** | ***β* = -0.10**  ***SE =* 0.02**  ***t* = -4.85 *p*<.001** |
| Male Sex: TimeFromBaseline | *β = -*0.06  *SE=* 0.03  *t* = -1.91 *p*=.06 | *β = -*0.01  *SE=* 0.03  *t* = -0.41 *p*=.68 | *β = -*0.04  *SE =* 0.03  *t* = -1.52 *p*=.13 | *β = -*0.03 *SE=* 0.03  *t* = -1.21 *p*=.23 | *β = -*0.03  *SE =* 0.02  *t* = -1.33 *p*=.18 | ***β = -*0.07**  ***SE=* 0.02**  ***t* = -3.34 *p<*.001** |
| MaleSex: SCD Classification: TimeFromBaseline | ***β* = 0.16**  ***SE =* 0.06**  ***t* = 2.70 *p*<.001** | *β* = 0.13  *SE =* 0.06  *t* = 1.96 *p=*.05 | ***β* = 0.14**  ***SE =* 0.06**  ***t* = 2.51 *p=*.012** | ***β* = 0.11**  ***SE =* 0.05**  ***t* = 2.27 *p=*.023** | ***β* = 0.12**  ***SE =* 0.04**  ***t* = 2.80 *p*<.001** | ***β* = 0.09**  ***SE =* 0.04**  ***t* = 2.28 *p=*.022** |
| **Contrasts** |  |  |  |  |  |  |
| SCD Classification: TimeFromBaseline (males) | *β = -*0.01  *SE =* 0.05  *t* = -0.24  *p=* .81 | *β = -*0.05  *SE =* 0.06  *t* = -0.83 *p=*.41 | *β = -*0.03  *SE =* 0.05  *t* = -0.55 *p=*.58 | *β =* 0.02  *SE=* 0.04  *t* = 0.48 *p=*.63 | *β =* 0.01  *SE =* 0.04  *t* = 0.38 *p=*.71 | *β < -*0.01  *SE=* 0.03  *t* = -0.22 *p=*.83 |

Supplemental Table 1: Linear mixed effects model including time from baseline as a random slope

*Notes:* Bolded values are results that remained significant after FDR correction. The additional contrast was completed to examine the difference in rate of change for male SCD+ vs. male SCD-.
